# Point‐of‐care HIV maternal viral load and early infant diagnosis testing around time of delivery at tertiary obstetric units in South Africa: a prospective study of coverage, results return and turn‐around times

**DOI:** 10.1002/jia2.25487

**Published:** 2020-04-23

**Authors:** Tendesayi Kufa, Ahmad H Mazanderani, Gayle G Sherman, Aurélie Mukendi, Tanya Murray, Faith Moyo, Karl‐Günter Technau, Sergio Carmona

**Affiliations:** ^1^ Centre for HIV and STIs National Institute for Communicable Diseases Johannesburg South Africa; ^2^ Faculty of Health Sciences School of Public Health University of the Witwatersrand Johannesburg South Africa; ^3^ Department of Medical Virology Faculty of Health Sciences University of Pretoria Pretoria South Africa; ^4^ Paediatric HIV Diagnostics Wits Health Consortium Pty Ltd Johannesburg South Africa; ^5^ Department of Paediatrics and Child Health Faculty of Health Sciences University of the Witwatersrand Johannesburg Johannesburg South Africa; ^6^ Department of Paediatrics Faculty of Health Sciences Empilweni Services and Research Unit Rahima Moosa Mother and Child Hospital University of the Witwatersrand Johannesburg South Africa; ^7^ National Priority Programmes National Health Laboratory Service Johannesburg South Africa; ^8^ Department of Molecular Medicine and Haematology Faculty of Health Sciences School of Pathology University of the Witwatersrand Johannesburg South Africa

**Keywords:** diagnostics, point‐of‐care, testing, viral load monitoring, viral suppression, vertical transmission, Africa

## Abstract

**Introduction:**

Maternal viral load monitoring (mVL) and early infant diagnosis (EID) are necessary to achieve elimination of mother‐to‐child transmission of HIV. Point‐of‐care testing can achieve better outcomes compared to centralized laboratory testing (CLT). We describe the first implementation of point‐of‐care (POC) mVL and EID testing around delivery at four high volume tertiary obstetric units (TOUs) in Gauteng, South Africa.

**Methods:**

Prospective study of pregnant women living with HIV (WLHIV) and their infants. During the period 1 June 2018 to 31 March 2019, routine staff collected blood specimens from women and their infants around delivery. Specimen collection occurred throughout the week while dedicated POC operators, conducted testing during working hours on weekdays. Descriptive statistics and multivariable Poisson regression with robust error variance were used to describe outcomes and associated factors. Outcomes determined were (i) coverage of mVL and EID testing defined as a proportion of live births to WLHIV admitted at each facility (ii) results returned prior to discharge (iii) turn‐around time (TAT) and iv) performance of POC testing compared to CLT.

**Results:**

In total, 8147 live births to pregnant WLHIV were recorded in the implementation period. Of these, 2912 mVL and 5074 EID specimens were included in the analysis, with 131 (4.5%) mVL and 715 (14.1%) EID specimens having initial invalid/error results. Overall coverage of POC mVL and EID testing was 35.6% (range 20.9% to 60.1%) and 61.9% (range 47.0% to 88.0%) respectively. Proportions of POC tested mothers and infants with results returned prior to discharge were 74.3% (range 39.0% to 95.7%) and 73.0% (range 50.0 to 97.9%). Return of results was independently associated with TOU, after‐hours specimen collection, having an initial invalid or error result and period of implementation. Overall TAT for specimens collected from mother‐infant pairs where both had POC testing, during weekdays was longer for EID compared to mVL testing (median 3.3 hours vs. 2.9 hours, *p*‐value sign test <0.001). POC results were comparable to those from laboratory testing.

**Conclusion:**

Accurate and timely POC mVL and EID testing around delivery was implemented with variable success across TOUs. Further scale up would need to address health system factors at facility level and high analytical error rates.

## 1 Introduction

The prevention of mother‐to‐child transmission (PMTCT) of HIV has been one of the most successful public health interventions in South Africa. The proportion of pregnant women living with HIV (WLHIV) who knew their HIV status at antenatal booking had exceeded 95%, with more than 95% of pregnant WLHIV on antiretroviral therapy (ART) prior to delivery by end of 2017 [1]. The intra‐uterine and intrapartum transmission rates decreased from >20% in 2003 [2], to 1.5% in 2017 [3]. However, this progress is insufficient to meet the targets for elimination of mother‐to‐child transmission of HIV (eMTCT).While the World Health Organisation (WHO) set the 2020 bronze tier target for elimination in high HIV prevalence settings at <750 new paediatric HIV infections per 100,000 live‐births, an estimated >1000 infants per 100,000 live births are infected each year in South Africa [4, 5].

Achieving maximum viral suppression throughout pregnancy and post‐partum periods is necessary to meet global elimination targets. Maternal viraemia at delivery has been associated with HIV transmission from mother to child [6]. South Africa delivers antenatal care through 4300 facilities nationally with access to centralized laboratory HIV viral load and early infant diagnosis (EID) testing. (National Department of Health, DHIS 2016) Measuring maternal viral loads (mVL) around the time of delivery may help identify mothers at risk of transmission and allow identification of infants eligible for high‐risk prophylaxis to further reduce risk of post‐natal transmission [6, 7]. Universal birth testing of HIV‐exposed neonates by point‐of‐care (POC) EID testing allows result return before discharge with immediate identification of *in utero*‐infected neonates at risk of rapid disease progression, for early ART initiation. Additionally, result return to the majority of HIV‐uninfected neonates maybe quicker and simplified in comparison to centralized laboratory testing (CLT).

POC VL testing improves turn‐around time (TATs) and retention in care among adults on ART [8], while POC EID testing reduces TATs and time to ART initiation in infants [9, 10, 11, 12, 13, 14]. Both have potential to reduce morbidity and mortality among pregnant WLHIV and their infants. Two POC VL and two POC EID assays have been prequalified by WHO. Cepheid’s Xpert HIV‐1 quantitative and HIV‐1 qualitative assay (Cepheid, Sunnyvale, CA, USA) [15, 16], as well as Abbott’s m‐Pima HIV‐1/2 VL and q‐HIV‐1/2 Detect (Abbott, Chicago, IL, USA) [17, 18], had comparable performance to standardized laboratory assays in multiple settings [9, 12, 19, 20, 21]. However, there is limited data on routine scale up POC testing for WLHIV and their infants.

Most women (96%) in South Africa deliver in approximately 700 obstetric units nationally [22], representing potential focal points for POC testing. We describe implementation of POC VL and EID testing around the time of delivery at four tertiary obstetric units (TOUs) in Gauteng Province, South Africa. We determine outcomes of POC testing implementation with respect to coverage, result return before discharge, TATs and performance compared to CLT. We hypothesized that TAT for both mVL and EID POC testing would be shorter compared to CLT, resulting in greater proportions of WLHIV and infants with results returned before discharge. We also hypothesized that the performance of POC testing would be comparable to that of CLT.

## 2 Methods

### 2.1 Setting

This implementation study was conducted at four high volume TOUs in Gauteng during the period 1 June 2018 to 31 March 2019. The TOUs were located in Johannesburg Regions B, D, F, and in Tshwane district. All had average monthly total number of live births of 520 to 1650 in the preceding year, of which 119 to 353 were to pregnant WLHIV and delivered pregnant women referred from lower level obstetric units in their catchment areas. During implementation, routine VL testing around the time of delivery was a newly introduced practice while EID testing at birth for all HIV‐exposed infants had been established practice since 2015.

### 2.2 Design and population

Prospective study of pregnant or early post‐partum WLHIV admitted to labour or post‐delivery wards and their new‐born infants until return of results or discharge.

### 2.3 Procedures

#### 2.3.1 Specimen collection

All WLHIV admitted to labour or postnatal wards at the four TOUs during the study period were offered POC VL and or birth PCR testing by routine staff. To be eligible for enrolment and specimen collection for the study, WLHIV and or their infants had to be admitted in labour or postnatal wards and be willing to provide verbal consent. For both WLHIV and infants, two specimens were collected – one for POC and the other for CLT. Specimens were collected by doctors and nurses as part of their routine duties. Non‐study patients could access HIV EID and VL POC testing where clinically indicated and these were labelled “miscellaneous.”

#### 2.3.2 POC testing

POC testing was conducted by a dedicated POC operator working in a designated POC testing room. While specimen collection took place throughout the week including weekends and after‐hours at all but one TOU (Johannesburg Region B), testing took place during working hours – 08:00 to 16:00 – on weekdays only. During the first three months of implementation, all specimens collected were processed and tested while afterwards only weekday specimens and those weekend specimens which had a reasonable probability of results being returned were tested. At two of the busier TOUs (Johannesburg region B and D), two dedicated counsellors hired through the study assisted with return of results and post‐test counselling before discharge.

For POC VL testing, Xpert™ HIV‐1 VL was used while POC EID testing was conducted using either the Xpert™ HIV‐1 Qual or the m‐PIMA HIV‐1/2 Detect assays. Upon receiving appropriate samples, POC operators entered specimen details into the instruments’ information management system and tested the specimens according to manufacturers’ specifications and sample volumes. Mothers and infants whose results were reported as error, invalid or no result had the test repeated on the same sample and if there was still no conclusive result, a second sample was collected as soon as possible after the result. Infants with a positive POC EID result were repeat tested on the same assay using the same sample to ensure reproducibility, and a second sample was requested for confirmatory testing on the alternate EID assay. Additional information on specimen collection, testing procedures and quality assurance are provided in Supplementary Document SD1.

#### 2.3.3 Return of results

POC operators printed results and gave them to TOU staff or counsellors for post‐test counselling. High maternal VL and positive EID results were automatically sent to the clinicians responsible for routine care by SMS for action. WLHIV who had high VLs were prescribed enhanced adherence counselling and repeat VL after three months while their EID negative infants were offered high‐risk prophylaxis, consisting of either (i) daily zidovudine (AZT) and nevirapine (NVP) for six weeks OR (ii) daily NVP for 12 weeks. Virally suppressed (VL < 1000 copies/mL) WLHIV were counselled on maintaining viral suppression and their EID negative infants offered low‐risk prophylaxis. All mothers of EID negative infants were counselled about further testing at 10 weeks of age. WLHIV with EID positive infants at birth were counselled and referred to the paediatrics department for ART initiation after a second sample drawn for confirmatory POC EID and CLT.

### 2.4 Data entry and management

Five different data sources were used to collect data required for the analyses of outcomes. These were (i) specimen request forms, (ii) POC instrument information system (iii) POC results slip (iv) centralized laboratory information system and (v) District Health information system monthly reports. Table S2 describes these data sources and how they were used. Sources (i) (iv) provided data which were captured into a study‐specific REDCap^®^ database. A data coordinator checked completeness of data entries and supported POC operators with collecting good quality data.

### 2.5 Outcomes and analyses

At the end of implementation, data captured in REDCap^®^ were exported into Stata^®^ 14.2 for data cleaning and analysis. WLHIV or infants whose indications for testing were marked as “miscellaneous” were excluded from analysis as were EQA specimens. Descriptive statistics were used to describe the distribution of mVL and EID testing overall and across TOUs. Table 1 shows the four main outcomes were defined and analysed. Poisson regression with robust error variance was used to determine factors associated with return of results. Additional information on sensitivity analyses and multivariable models are included in Supplementary Document SD1.

**Table 1:** Description of outcomes and data analysis techniques

Outcome	POC test	Definition	Data analysis methods
Coverage of testing	a) VL	Number of WLHIV who had a POC VL test done as % of live births to WLHIV during implementation period (Minimum coverage). Maximum coverage^a^	Coverage was measured on the number of unique WLHIV regardless of the number of tests done on each woman. Proportions determined overall and across TOUs categories.
	b) EID	Number of HIV‐exposed infants who had POC EID test done as % of live births to WLHIV during the implementation period (Minimum coverage). Maximum coverage^a^	Coverage was measured on the number of unique HIV‐exposed infants regardless of number of tests done on each infant. Proportions were determined overall and across TOUs.
Results return before discharge	a) VL	% of WLHIV tested and had their POC results returned before discharge. Minimum % results returned were calculated using the number of live births to WLHIV during implementation period as denominator. Maximum % results returned^a^	Results return was measured on the number of unique WLHIV or HIV‐exposed infants regardless of the number of tests done on each woman. Proportions determined overall and across categories. χ^2^ tests used to compare differences across categories. Multi‐variable Poisson regression with robust error variance was used to determine sample and TOU‐level factors associated with return of results. All available variables were included in models *a priori.* ROC curves were used to test for significance difference between models with interaction terms and those without.
	b) EID	% of infants tested who had their POC results returned before discharge. Minimum % results returned were calculated using the number of live births to WLHIV during implementation period as denominator. Maximum % results returned^a^	
Turn‐around time (TAT)	VL and EID	Duration of time from specimen collection to return of results.	This was measured on all specimens tested, resulted and returned to WLHIV or HIV‐exposed infants. Medians and interquartile ranges overall and across categories. K‐test for equality of medians used to determine differences across categories. These were calculated for specimens with a documented return of result. The sign‐test was used to compare the population level median TAT for POC VL testing versus POC EID testing for mothers‐infant pairs who both had POC testing.
Agreement between POC results and laboratory results	a) VL	Limits of agreement between POC VL results and centralized laboratory VL results. Agreement beyond chance for VL results below limit of detection, VL > 50 copies/mL, VL > 1000 copies/mL	Bland‐Altman analysis. Kappa Statistic, sensitivity and specificity of POC testing compared to CLT
	b) EID	Agreement beyond chance for positive or negative EID results	Kappa Statistic, sensitivity and specificity of POC testing compared to CLT

EID, early infant diagnosis; mVL, maternal HIV viral load; POC, point‐of‐care test.

a The outcomes of “maximum coverage of testing” and “maximum % results returned”, were calculated by excluding live births to WLHIV delivered during weekends or on public holidays.

### 2.6 Ethical considerations

The protocol for this implementation study was approved by the University of the Witwatersrand Human Subjects Research Ethics Committee (M1711115) and the University of Pretoria Research Ethics Committee (50/2018). Approvals were also obtained from the national and provincial Departments of Health as well as from the management at the tertiary hospitals where the obstetric units are situated. Verbal consent to perform POC testing was obtained from admitted women. Written informed consent for research purposes was waived to facilitate measuring routine implementation. Because of this waiver, patient details other than those required for return of results could not be collected. As the REDCap^®^ database collected personal identifiable information such as name, surname, folder number and specimen barcodes required for return of results and linkage to centralized laboratory database, only POC operators and key study staff had access to the database.

## 3 Results

### 3.1 Summary of testing

**Figure 1:**
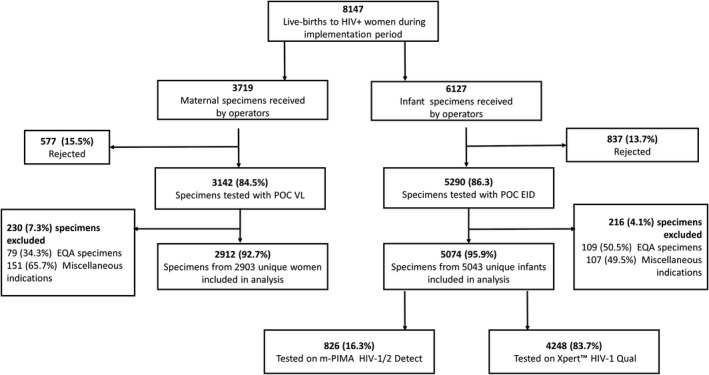
Summary of POC VL and EID testing.

Throughout the implementation period, all TOUs provided both POC VL and EID services alongside routine CLT. A total of 8147 live births to WLHIV were recorded across the TOUs – range 1177 at Tshwane District – 3334 at Johannesburg Region D. During this time, 3719 specimens for POC VL testing were received, of which 3142 (84.5%) were tested and 2912 (92.7%) specimens from 2903 unique women were included in this analysis (Figure 1). For POC EID, 6127 specimens were received, of which 5290 (86.3%) were tested and 5074 (95.9%) from 5043 unique infants were included. Indications for miscellaneous VL or EID testing included routine patient VL monitoring, diagnostic dilemmas and needles stick injuries. For POC VL, the majority of specimens collected but not tested were so because of insufficient volumes while for EID this was because of clotted samples, insufficient volumes and administrative errors. An invalid/error result on initial testing was reported in 131/2912 (4.5%) VL and 715/5074 (14.1%) EID specimens tested – 657 on Xpert (invalid/error rate of 15.5%) and 58 on m‐PIMA (invalid/error rate of 7.0%). Of the 131 initial VL invalid/ error results, 105 (80.2%) were errors while 26 (19.8%) were invalids/ no results. For POC EID testing of the 715 initial invalid/error results, 581 (81.3%) were invalid, 310 (43.4%) were reported in Quarter 2 (July September 2018); 473 (61.2%) occurred equally at Johannesburg D and F TOUs and 359 (50.2%) occurred with cartridges from three lots.

### 3.2 Coverage of testing and return of results

Using live births to WLHIV as the denominator, minimum overall coverage of POC VL testing was 35.6% (range 20.9% to 60.1% across TOUs) while minimum overall coverage EID was 61.9% (range 47.0% to 88.0% across TOUs) (Table 2). Using denominators adjusted for non‐testing of specimens collected on weekend and public holidays, maximum overall coverage was 50.3% (range 29.9% to 92.4% across TOUs) for POC mVL testing and 87.5% (range 63.5% to 100% across TOUs) for EID testing.

**Table 2:** Coverage of testing and return of results

	VL					EID				
	Jhb F	Jhb D	Tshwane	Jhb B	All	Jhb F	Jhb D	Tshwane	Jhb B	All
Infants born to WLHIV	1543	3324	1177	2103	8147	1543	3324	1177	2103	8147
Infants born to WLHIV (excluding weekends/ public holidays)	1080	2460	859	1367	5766	1080	2460	859	1367	5766
Number of unique WLHIV OR infants tested	323	743	574	1263	2903	1038	1561	1036	1408	5043
% Coverage of testing (Minimum)	20.9	22.4	48.8	60.1	35.6	67.3	47.0	88.0	67.0	61.9
% Coverage of testing (Maximum)	29.9	30.2	66.8	92.4	50.3	96.1	63.5	100	100	87.5
Infants whose mothers had POC VL testing	–	–	–	–	–	142	233	219	1118	1712
% infants whose mothers had POC VL testing	–	–	–	–	–	13.7	14.9	21.1	79.4	33.9
Number with documented results returned before discharge	274	290	383	1209	2156	801	780	721	1379	3681
% Results returned (Minimum)	17.8	8.7	32.5	57.5	26.5	51.9	23.5	61.3	65.6	45.2
% Results returned (Maximum)	84.8	39.0	66.7	95.7	74.3	77.2	50.0	69.6	97.9	73.0
Number with VL ≥ 1000 copies/mL/EID positive	58	156	154	254	622	7	15	24	19	65
% VL ≥ 1000 copies/mL OR EID positive	18.0	21.0	26.8	20.1	21.4	0.7	1.0	2.3	1.4	1.3

Jhb B, Johannesburg B; Jhb D, Johannesburg D; Jhb F, Johannesburg F; EID, early infant diagnosis; POC, point‐of‐care; VL, HIV viral load.

The proportion of unique WLHIV tested by POC VL who had mVL results returned prior to discharge was 74.3% (range 39.0 96.2% across TOUs) while that of unique infants tested by POC EID was 73% (range 50.0 95.7% across TOUs). The minimum proportion of mVL POC results returned ranged from 8.7% to 57.5% while that for EID ranged from 23.5 65.6% (Table 2). Failure to return results was due to WLHIVand/or infants being discharged prior to POC results becoming available.

For both POC VL and EID testing, the relative risk associated with results not being returned were higher at Johannesburg D and F and Tshwane compared to Johannesburg B, for specimens collected after‐hours and when the initial POC test yielded an error/invalid result (Table 3). For both VL and EID POC testing, the risk of results not being returned was lower with successive quarters. For POC VL testing, the risk of results not being returned was independently higher for specimens with high viral loads results. The overall proportion of infants who were POC EID positive at birth (intra‐uterine transmission rate) was 1.3% (range 0.7% to 2.3%).

**Table 3:** Factors associated with no results return before discharge for both VL and EID testing

Variable/category	VL (N = 2903)					EID (N = 5043)				
	n/N	Univariable RR (95% CI)	*p*‐value	Multivariable aRR (95% CI)	*p*‐value	n/N	Univariable RR (95% CI)	*p*‐value	Multivariable aRR (95% CI)	*p*‐value
TOU^a^										
Johannesburg F	49/313	3.55 (2.46 to 5.12)	<0.001	3.58 (2.13 to 6.04)	<0.001	237/1038	11.08 (7.60 to 16.16)	<0.001	8.01 (4.96 to 12.91)	<0.001
Johannesburg D	453/743	14.26 (10.92 to 18.63)	<0.001	17.55 (12.92 to 23.84)	<0.001	781/1561	24.29 (16.87 to 34.94)	<0.001	22.71 (15.00 to 34.41)	<0.001
Tshwane	191/574	7.78 (5.85 to 10.35)	<0.001	7.51 (5.33 to 10.59)	<0.001	315/1036	14.76 (10.18 to 21.41)	<0.001	14.01 (9.18 to 21.49)	<0.001
Johannesburg B	15/1263	1.00		1.00		29/1408	1.00		1.00	
Quarter of enrolment										
June 18	81/281	1.00		1.00		133/384	1.00		1.00	
July to September 18	260/905	1.00 (0.81 to 1.23)	0.975	1.02 (0.86 to 1.21)	0.845	688/1725	1.15 (0.99 to 1.34)	0.064	0.89 (0.79 to 1.01)	0.066
October to December 18	202/849	0.83 (0.66 to 1.03)	0.087	0.68 (0.57 to 0.81)	<0.001	292/1430	0.60 (0.50 to 0.70)	<0.001	0.50 (0.43 to 0.57)	<0.001
January to March 19	204/868	0.82 (0.65 to 1.06)	0.068	0.59 (0.49 to 0.70)	<0.001	249/1504	0.48 (0.40 to 0.57)	<0.001	0.38 (0.32 to 0.44)	<0.001
Timing of specimen collection^a^										
Weekday workhours	284/1859	1.00		1.00		545/2619	1.00		1.00	
Weekday after‐hours^β^	353/772	2.99 (2.62 to 3.41)	<0.001	5.52 (2.92 to 10.41)	<0.001	546/1694	1.55 (1.40 to 1.71)	<0.001	3.62 (1.14 to 11.54)	0.030
Weekend	110/272	2.65 (2.21 to 3.17)	<0.001	1.43 (1.08 to 1.90)	0.012	271/730	1.78 (1.58 to 2.01)	<0.001	1.17 (0.95 to 1.43)	0.143
POC machine										
qHIV‐1/2 detect						135/825	1.00		1.00	
Xpert HIV1/2 qual						1227/4218	1.78 (1.51 to 2.09)	<0.001	0.93 (0.79 to 1.09)	0.373
Initial test invalid/error										
No	657/2772	1.00		1.00		906/4332	1.00		1.00	
Yes	90/131	2.90 (2.54 to 3.31)	<0.001	2.17 (1.90 to 2.49)	<0.001	456/711	3.07 (2.83 to 3.32)	<0.001	2.69 (2.50 to 2.90)	<0.001
VL ≥ 1000 copies/mL										
No	573/2281	1.00		1.00		–	–	–	–	–
Yes	174/622	1.11 (0.96 to 1.29)	0.149	1.16 (1.03 to 1.32)	0.018	–	–	–	–	–
Mother did not have POC mVL										
No	–	–	–	–	–	1183/3331	1.00		1.00	
Yes	–	–	–	–	–	179/1712	3.40 (2.94 to 3.93)	<0.001	1.29 (1.12 to 1.47)	<0.001
EID PCR positive										
No	–	–	–	–	–	12/65	1.00		1.00	
Yes						1350/4978	1.47 (0.88 to 2.45)	0.142	1.12 (0.68 to 1.86)	0.655

β‐specimens collected before 07:00 hours and after 15:00 hours were considered to have been collected after hours. aRR, adjusted relative risk; CI, confidence interval; EID, early infant diagnosis; RR, relative risk; POC, point‐of‐care; TOU, tertiary obstetric unit; VL, viral load.

^a^ Both models allowed for interaction between timing off specimen collection and TOU: *p*‐value for interaction terms were 0.002 for mVL model and 0.034 for EID models respectively.

### 3.3 Turn‐around times

Overall median TATs for POC VL testing was 3.3 hours (IQR 2.2 to 18.2 hours), n = 2175 while that of EID POC testing was longer at 9.6 hours (IQR 2.9 to 22.6 hours), n = 3717. The proportion of specimens with TATs < 24 hours were 82.8% for POC VL testing compared to 77.6% for POC EID testing. For mother‐infant pairs where both mother and infant had POC testing, overall median TAT was longer for EID compared to VL; 3.3 hours (IQR 2.5 to 9.3 hours) versus 2.9 hours (IQR 2.1 to 7.4 hours), *p*‐value sign test <0.001). TATs were longer for specimens collected during the weekend compared to those collected during weekdays – 4.9 hours (IQR 2.4 26.2 hours) versus 3.2hours (IQR 2.2 16.6 hours) for POC VL and 33 hours (IQR 23 46 hours) versus 6.5 hours (IQR 2.8 to 19 hours) for POC EID testing‐ see Figure S1. In analyses excluding weekend specimens, the pre‐test period (time between specimen collection and obtaining a result from the instrument) accounted for the bulk of the TAT (Figure S2). POC pre‐test periods during weekdays were shorter compared to the CLT pre‐test periods for both mVL and EID testing (see Figure S3).TATs were longer at Johannesburg D, Johannesburg F and Tshwane TOUs compared to Johannesburg B and for specimens collected after‐hours but shorter with successive quarters of enrolment for VL testing. For EID testing they were also longer at Johannesburg D, Johannesburg F and Tshwane TOUs compared to Johannesburg B, for specimens collected after‐hours and among specimens with an initial invalid/error result (Table 4).

**Table 4:** POC VL and EID testing turn‐around times by different categories measured on weekday specimens

Category	VL (N = 2012)		EID (N = 3251)	
	TAT in hours (median, IQR)	*p*‐value	TAT in hours (median, IQR)	*p*‐value
TOU				
Johannesburg F	11.9 (3.4 to 19.4)		14.2 (8.3 to 19.8)	
Johannesburg D	25.5 (15.5 to 34.8)	<0.001	22.5 (12.2 to 28.2)	<0.001
Tshwane	14.6 (3.5 to 22.0)		15.5 (4.3 to 24.7)	
Johannesburg B	2.5 (2.0 to 3.3)		2.8 (2.3 to 3.6)	
Quarter of enrolment				
June 18	3.8 (2.8 to 8.9)		7.8 (3.3 to 11.8)	
July to September 18	3.3 (2.4 to 8.4)	<0.001	6.1 (3.1 to 18.8)	0.809
October to December 18	2.8 (2.1 to 15.5)		6.6 (2.6 to 19.3)	
January to March 19	2.8 (2.0 to 18.0)		6.3 (2.6 to 20.2)	
Specimen collected after‐hours^a^				
No	2.6 (2.1 to 3.8)		3.2 (2.4 to 8.8)	
Yes	17 (11.8 to 30.0)	<0.001	14.6 (9.8 to 20)	<0.001
Mother/infant also tested by POC				
No	11.3 (2.8 to 23.4)		14 (4.5 to 22.9)	
Yes	2.8 (2.1 to 4.3)	<0.001	3.2 (2.5 to 6.7)	<0.001
Instrument type				
Xpert HIV1/2 Qual	**–**	–	6.7 (2.7 to 19.1)	
qHIV‐1/2 Detect	**–**	–	5.3 (3.0 to 18.8)	0.284
Initial test invalid or error				
No	3.1 (2.2 to 13.6)		6.3 (2.7 to 19.0)	
Yes	3.3 (2.4 to 10.0)	0.382	7.6 (3.7 to 20.2)	0.002
Test result (VL ≥ 1000/EID pos)				
No	3.1 (2.2 to 13.2)		6.3 (2.8 to 19.0)	
Yes	3.3 (2.3 to 14.0)	0.284	14.2 (2.7 to 21.8)	0.151

EID, early infant diagnosis test; IQR, interquartile range; POC, point‐of‐care; pos, positive; TAT, turn‐around time; VL, viral load.

a specimens collected before 07h00 and after 15h00 were considered to have been collected after hours.

Johannesburg B TOU demonstrated the best coverage, result return rate and TATs of all TOUs but differed from the other three TOUs in that the facility already had four years of experience with POC EID testing, having been a clinical trial site, and had additional staff at hand to support implementation.

### 3.4 Performance of POC testing compared to CLT

**Figure 2:**
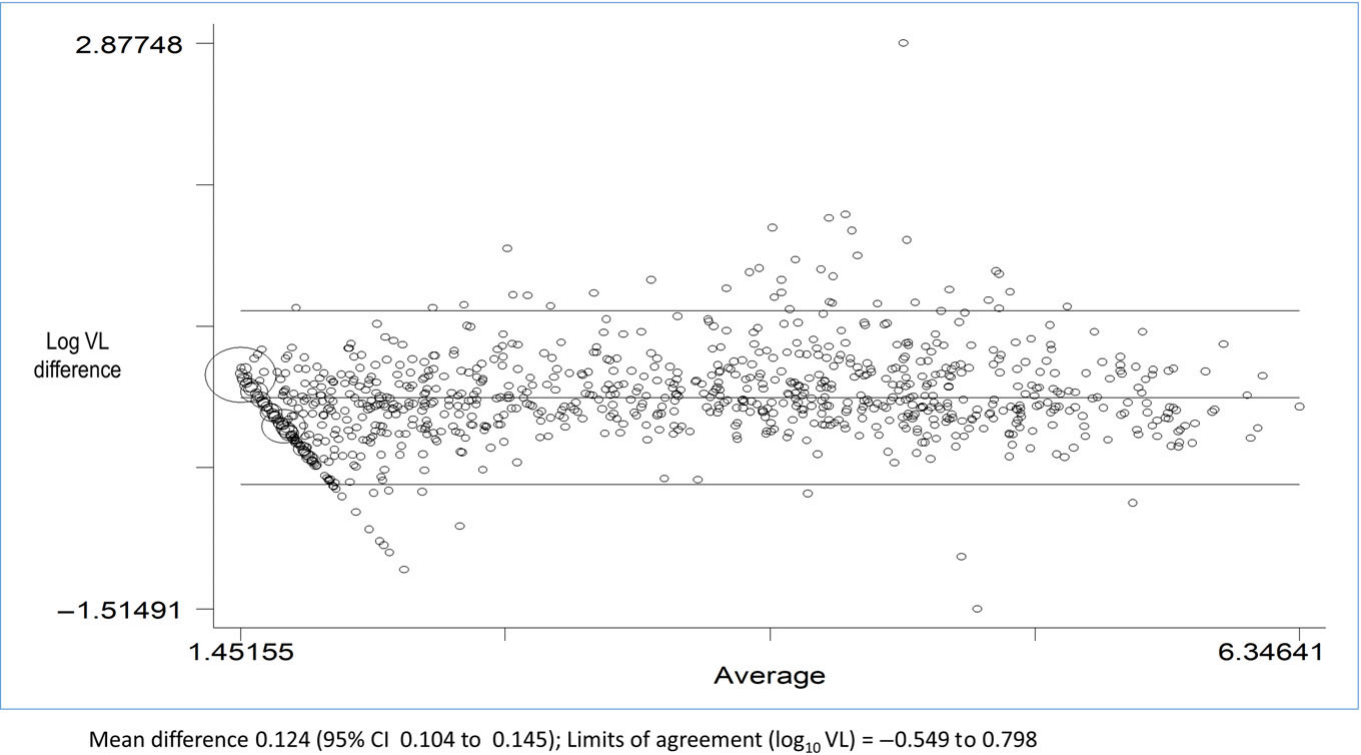
Bland Altman plot of agreement between POC VL and centralized laboratory VL measurements.

There was good agreement between results of both POC VL and EID testing with CLT. For all quantifiable and paired VL measurements (N = 2352), the limits of agreement were log_10_ VL 0.549 to log_10_ VL 0.798, (Figure 2) and the Spearman correlation coefficient was 0.95 (95% CI 0.95 to 0.96). At VL load thresholds of i) RNA detectable/ undetectable, ii) 50 to 1000 copies/mL and iii) >1000 copies/mL, POC and laboratory VL had percent agreements of i) 78.9% (95% CI 77.2 to 80.5, ii) 88% (95% CI 86.6 to 89.2%) and iii) 98.2% (95% CI 97.5 98.6%) respectively (see Table S3A).

For paired POC EID testing results (N = 4081), overall agreement for positive/negative results was 99.5% (95% CI 99.3 99.7 %) – see Table S3B,C,D. There were 10 (0.25%) infants who had discrepant POC and centralized laboratory results. Of these, five (50%) had HIV‐detected on POC EID testing but not by laboratory testing and five (50%) had HIV detected on laboratory EID testing but not by POC testing. Johannesburg B and D and Tshwane TOUs equally accounted for nine of the 10 infants who had discrepant POC and laboratory EID results. Of the five POC EID positive infants, three had repeat EID testing and two EID negative on follow‐up testing. On follow‐up of the five who were laboratory EID positive, one was confirmed HIV positive and started ART, the rest did not have repeat CLT.

## 4 Discussion

We described implementation of POC VL and EID testing around delivery at four high volume TOUs in Gauteng province, South Africa. POC VL and EID testing was implemented with variable success across the TOUs. POC EID testing coverage was higher than POC VL testing coverage but were at best 88% and 50% respectively. Result return rates were similar between POC VL and EID testing and both varied by TOU, quarter of implementation, specimen collection after‐hours and initial test result being invalid/error. TAT varied greatly across TOUs. There was good agreement between paired POC and centralized laboratory results for both VL and EID testing.

Our study demonstrated feasibility of POC VL and EID testing around delivery but highlights the need for additional resources to achieve adequate testing coverage and result return. To the best of our knowledge, this is the first study describing implementation of both POC VL and EID testing for WLHIV and their infants around time of delivery. VL and EID POC testing at this time represents the potential for large proportions of WLHIV and their infants to access HIV testing by strategically placing POC instruments in the busiest delivery sites in South Africa. The benefits include identification of suboptimal viral suppression and the opportunity to decrease transmission risk of HIV in the peri‐ and post‐partum period by prescribing high‐risk infant prophylaxis and addressing maternal viraemia respectively. HIV‐exposed infants benefit from improved result return, linkage to care and early ART initiation among HIV‐infected infants [11]. EID POC testing also provides an opportunity for immediate return of negative birth test results, reassuring mothers and counselling them on future HIV testing requirements for their infants. An earlier study performed at Johannesburg B TOU, showed that linkage to care following centralized laboratory‐based birth testing was sub‐optimal and that delays in getting results to WLHIV could be a contributory factor [23].

Poorer coverage of mVL compared to POC EID testing may be attributable to EID testing at birth being established practice in obstetric units whilst maternal VL testing at delivery new. Consequently, routine staff at Johannesburg D and F and Tshwane TOUs required multiple reminders to collect POC VL testing samples over the first few months of implementation. The wide variation of the main outcomes across TOUs may suggest differences in underlying health system factors such as patient flow and capacity of routine staff. For effective scale up of POC testing in similar settings, determining optimal patient flows and placement of instruments to achieve best coverage, return of results and shortest TATs without burdening current staff is required. Improvements in proportions of results returned before discharge, and reduced TATs, during implementation study likely reflects improvements in specimen collection, processing and result‐return procedures over time. The lower proportion of results returned and longer TATs for specimens with an initial invalid/error result was expected. Repeat testing on the same sample lengthened the TAT and reduced the likelihood of results being returned before discharge. Minimizing the proportion of tests reported as error/invalid will improve TATs and result return. The high error/invalid rate with POC EID testing could partly explain longer TAT compared to POC VL testing. Higher volumes and proportions of weekday specimens collected after‐hours for POC EID testing (1743 (40.1%)) compared to POC VL testing (807 (30.6%)) could also account for longer TATs in the former when compared to the latter. The larger numbers of after‐hours EID specimens meant EID specimens waited longer before processing compared to VL specimens. The association of failure to return mVL results with high viral loads was unexpected. This could have been due to residual confounding not adequately adjusted for in the multivariable model. The higher proportion of results returned and associated shorter TATs among mothers and infants among mother‐infant pairs where both were tested using POC was unexpected but could be explained by prioritizing testing of infants whose mothers had high mVLs, either by the POC operators or by the health care providers.

The proportions with POC EID error/invalid results in our study were higher than proportions of up to 9.3% reported in other studies [9, 12, 20, 21, 24]. Among the EID error/invalid results, 50.2% were associated with cartridges belonging to three specific lots which may suggest faulty cartridges. Our study had higher error rates compared to earlier studies where POC EID testing was done four‐six weeks of age whereas our study offered POC testing at birth. Drawing blood at birth is challenging and associated with insufficient specimens. This could have contributed to the higher error rate. We cannot exclude operator error as a contributory cause, although training was provided continuously throughout implementation.

Our study had a number of limitations. First, to determine coverage of testing and return of results we used the number of live births to WLHIV determined from routine data as a proxy for (1) number of WLHIV admitted during the implementation period and (2) the number of HIV‐exposed infants. Use of this denominator, known to be undercounted, may have under‐estimated numbers of WLHIV or their infants eligible for testing thereby overestimating coverage. Efforts to collect denominator data were challenged by use of multiple recording and reporting tools across TOUs. Second, because written informed consent for research was waived, we could not collect additional patient level data such as maternal ART use or duration, data that would have been useful in interpretation of maternal viral load or infant EID results. POC implementation relied on routine TOU staff, and not study phlebotomists, to collect blood specimens. Since laboratory and POC testing was performed in parallel, the volume of blood required doubled and may have contributed to insufficient samples. Third, POC testing occurred during working hours. Although this was necessary because of funding constraints, it resulted in some WLHIV and their infants being missed. Any future scale up of POC VL and EID testing around time of delivery would need to consider after‐hours and weekend testing. Lastly, although cost data were collected during the study, findings from these data were beyond the scope of this analysis.

## 5 Conclusion

Our implementation showed that POC EID and mVL testing around time of delivery was feasible, accurate and could achieve high coverage in settings with additional resources. Further scale up needs to address health systems challenges, high error rates and after‐hours testing. Evaluations of the impact of POC VL and EID testing at delivery on improving postnatal maternal VL suppression and linkage to and retention in care as well as its effect on mother‐to‐child transmission of HIV – such as those planned in Zimbabwe and Mozambique [25, 26] are required to inform this scale up.

## Competing interest

TK received non‐financial support from Cepheid during the conduct of the study; K‐GT received grants from CHAI/UNITAID during the conduct of the study; all the other authors declared no conflict of interest.

## Authors’ contributions

SC, GGS and AHM designed the study. GGS, AHM, K‐GT, TM and AM oversaw data collection. TK and FM analysed the data. TK wrote the paper. All authors reviewed the manuscript and approved the final version for submission.

## Supporting information


**Supplementary Document SD1.** Supplementary methods
**Figure S1.** Turn‐around times for weekday and weekend specimens.
**Figure S2.** Turnaround times by the different components – weekday specimens.
**Figure S3.** Comparison of pre‐test POC and CLT pre‐test periods – weekday specimens.

**Table S1.** Differences in POC instruments and staff per site

**Table S2.** Data sources and management for point‐of‐care implementation outcomes evaluation
**Table S3.** **(A)** Performance of POC mVL testing compared to CTL. **(B)** Overall Performance of POC EID testing compared to CTL. **(C)** Performance of POC EID testing (Xpert HIV‐1/2 Qualitative) compared to CTL **(D)** Performance of POC EID testing (q‐HIV1/2 Detect) compared to CTL

## References

[1] Woldesenbet SA , Kufa T , Lombard C , Manda S , Ayalew K , Cheyip M , et al. The 2017 National Antenatal Sentinel HIV Survey, South Africa. Pretoria, Republic of South Africa: National Department of Health; 2019 [cited 2019 Oct 11]. Available from: https://www.nicd.ac.za/publications/special-publications/

[2] Sherman GG , Lilian RR , Bhardwaj S , Candy S , Barron P . Laboratory information system data demonstrate successful implementation of the prevention of mother‐to‐child transmission programme in South Africa. S Afr Med J. 2014;104(3):2078–5135. 10.7196/samj.7598 24893499

[3] Goga A , Chirinda W , Ngandu NK , Ngoma K , Bhardwaj S , Feucht U , et al. Closing the gaps to eliminate mother‐to‐child transmission of HIV (MTCT) in South Africa: understanding MTCT case rates, factors that hinder the monitoring and attainment of targets, and potential game changers. S Afr Med J. 2018;108(3a):2078–5135.

[4] World Health Organization . Global guidance on criteria and processes for validation: elimination of mother‐to‐child transmission of HIV and syphilis. 2nd edn Geneva, Switzerland: World Health Organization; 2017.

[5] Haeri Mazanderani A , Macleod WB , Bor J , Sherman GG . Age at HIV diagnosis within South Africa’s Early Infant Diagnosis Program, 2010–2015. Conference on Retroviruses and Opportunistic Infection. 4–7 March 2018; Boston, MA.

[6] Myer L , Phillips TK , McIntyre JA , Hsiao NY , Petro G , Zerbe A , et al. HIV viraemia and mother‐to‐child transmission risk after antiretroviral therapy initiation in pregnancy in Cape Town, South Africa. HIV Med. 2017;18(2):80–8. 27353189 10.1111/hiv.12397

[7] Lesosky M , Glass T , Mukonda E , Hsiao NY , Abrams EJ , Myer L . Optimal timing of viral load monitoring during pregnancy to predict viraemia at delivery in HIV‐infected women initiating ART in South Africa: a simulation study. J Int AIDS Soc. 2017;20 Suppl 7:e25000. 10.1002/jia2.25000 PMC5978661 29171179

[8] Drain PK , Dorward J , Violette LR , Quame‐Amaglo J , Thomas KK , Samsunder N , et al. Point‐of‐care HIV viral load testing combined with task shifting improve treatment outcomes (STREAM): findings from an an open‐label, non‐inferiority, randomised controlled trial. Lancet HIV. 2020;7(4):e229–e37. 32105625 10.1016/S2352-3018(19)30402-3 PMC7183312

[9] Sabi I , Mahiga H , Mgaya J , Geisenberger O , Kastner S , Olomi W , et al. Accuracy and operational characteristics of Xpert human immunodeficiency virus point‐of‐care testing at birth and until week 6 in human immunodeficiency virus‐exposed neonates in Tanzania. Clin Infect Dis. 2019;68(4):615–22. 29961841 10.1093/cid/ciy538 PMC6355822

[10] Spooner E , Govender K , Reddy T , Ramjee G , Mbadi N , Singh S , et al. Point‐of‐care HIV testing best practice for early infant diagnosis: an implementation study. BMC Public Health. 2019;19(1):731. 31185962 10.1186/s12889-019-6990-z PMC6560857

[11] Bianchi F , Cohn J , Sacks E , Bailey R , Lemaire JF , Machekano R , et al. Evaluation of a routine point‐of‐care intervention for early infant diagnosis of HIV: an observational study in eight African countries. Lancet HIV. 2019;6(6):e373–81. 30987937 10.1016/S2352-3018(19)30033-5

[12] Opollo VS , Nikuze A , Ben‐Farhat J , Anyango E , Humwa F , Oyaro B , et al. Field evaluation of near point of care Cepheid GeneXpert HIV‐1 Qual for early infant diagnosis. PLoS ONE. 2018;13:e0209778. 30589900 10.1371/journal.pone.0209778 PMC6307732

[13] Jani IV , Meggi B , Loquiha O , Tobaiwa O , Mudenyanga C , Zitha A , et al. Effect of point‐of‐care early infant diagnosis on antiretroviral therapy initiation and retention of patients. AIDS. 2018;32(11):1453–63. 29746301 10.1097/QAD.0000000000001846

[14] Mwenda R , Fong Y , Magombo T , Saka E , Midiani D , Mwase C , et al. Significant patient impact observed upon implementation of point‐of‐care early infant diagnosis technologies in an observational study in Malawi. Clin Infect Dis. 2018;67(5):701–7. 29490026 10.1093/cid/ciy169 PMC6093992

[15] WHO Prequalification of In Vitro Diagnostics . WHO PQ public report: Xpert^®^ HIV‐1 Viral Load with GeneXpert^®^ Dx, GeneXpert^®^ Infinity48, GeneXpert^®^ Infinity‐48s and GeneXpert^®^ Infinity‐80. 2017 [cited 2020 Mar 17]. Available from: https://www.who.int/diagnostics_laboratory/evaluations/pq-list/hiv-vrl/170720_final_pq_report_pqdx_0192_0193_0194_0195_070-00.pdf?ua=1

[16] WHO Prequalification of In Vitro Diagnostics . WHO PQ public report: Xpert^®^ HIV‐1 Qual Assay WHO reference number: PQDx 0259–070‐00. 2016 [cited 2020 Mar 17]. Available from: https://www.who.int/diagnostics_laboratory/evaluations/pq-list/hivvrl/160613PQPublicReport_0259-0700-00_XpertQualHIV_v2.pdf

[17] WHO Prequalification of In Vitro Diagnostics . WHO PQ public report: Alere™ q HIV‐1/2 Detect. 2016 [cited 2020 Mar 17]. Available from: https://www.who.int/diagnostics_laboratory/evaluations/pq-list/hiv-vrl/160613PQPublicReport_0226-032-00AlereHIVDetect_v2.pdf

[18] WHO Prequalification of In Vitro Diagnostics . WHO PQ public report: m‐PIMA HIV‐1/2 VL. 2019 [cited 2020 Mar 17]. Available from: https://www.who.int/diagnostics_laboratory/evaluations/pq-list/190408_pqdx_0359_032_00_pqpr_mpima.pdf

[19] Ibrahim M , Moyo S , Mohammed T , Mupfumi L , Gaseitsiwe S , Maswabi K , et al. High sensitivity and specificity of the Cepheid Xpert^®^ HIV‐1 Qualitative Point‐of‐Care test among newborns in Botswana. J Acquir Immune Defic Syndr.2017;75(5):e128–31. 28350554 10.1097/QAI.0000000000001384 PMC5503760

[20] Murray TY , Sherman GG , Nakwa F , MacLeod WB , Sipambo N , Velaphi S , et al. Field evaluation of performance of Alere and Cepheid qualitative HIV assays for pediatric point‐of‐care testing in an academic hospital in Soweto, South Africa. J Clin Microbiol. 2017;55(11):3227–35. 28855305 10.1128/JCM.01021-17 PMC5654906

[21] Bwana P , Ageng’o J , Mwau M . Performance and usability of Cepheid GeneXpert HIV‐1 qualitative and quantitative assay in Kenya. PLoS ONE. 2019;14:e0213865. 30901343 10.1371/journal.pone.0213865 PMC6430374

[22] National Department of Health (NDoH), Statistics South Africa, South African Medical Research Council (SAMRC), and ICF . South Africa Demographic and Health Survey 2016. Pretoria, South Africa, and Rockville, Maryland: NDoH, Stats SA, SAMRC, and ICF; 2019.

[23] Technau K‐G , Strehlau R , Patel F , Shiau S , Burke M , Conradie M , et al. 12‐month outcomes of HIV‐infected infants identified at birth at one maternity site in Johannesburg, South Africa: an observational cohort study. Lancet HIV. 2018;5(12):e706–14. 30416043 10.1016/S2352-3018(18)30251-0 PMC6336389

[24] Technau K‐G , Kuhn L , Coovadia A , Murnane PM , Sherman G . Xpert HIV‐1 point‐of‐care test for neonatal diagnosis of HIV in the birth testing programme of a maternity hospital: a field evaluation study. Lancet HIV. 2017;4(10):e442–8. 28711526 10.1016/S2352-3018(17)30097-8 PMC5623143

[25] ClinicalTrials.gov . Identifier NCT00287391, Impact of Point‐of‐Care (POC) Viral Load (VL) Testing on Ensuring Appropriate Management of Viremia During Pregnancy to Prevent Vertical Transmission: an Observational Difference‐in‐difference Cohort Study. 19^th^ August 2019 [cited 3 Jan 2020]. Available from: https://clinicaltrials.gov/ct2/show/study/NCT04048629

[26] ClinicalTrials.gov . Identifier NCT04032522, Neonatal HIV Early Infant Diagnosis (EID) Versus Standard of Care EID – Long Term Impact on inFant hEalth: a Feasibility Study of point‐of Care Testing at Birth Versus at 6 Weeks of Age, on the Uptake of ART and Infant Prophylaxis, and on Rates of Infant Survival, Morbidity and Retention in Care. 25 July 2019 [cited 3 Jan 2020]. Available from: https://clinicaltrials.gov/ct2/show/study/NCT04032522

