# Supplementary material for: Point‐of‐care HIV maternal viral load and early infant diagnosis testing around time of delivery at tertiary obstetric units in South Africa: a prospective study of coverage, results return and turn‐around times
Source: J Int AIDS Soc. 2020 Apr 23;23(4):e25487. doi: 10.1002/jia2.25487 (PMC7180267; doi:10.1002/jia2.25487)

**Supplementary figures**

**Figure S1: Turn-around times for weekday and weekend specimens**


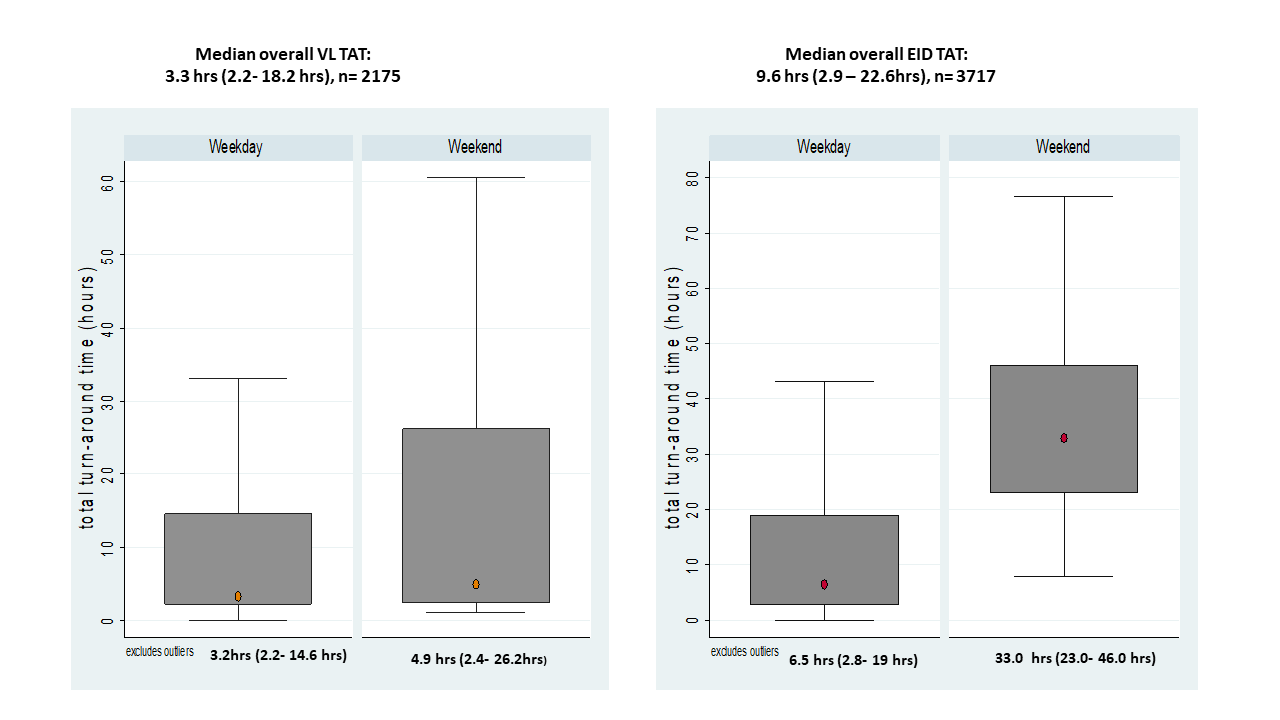


**Figure S2: Turnaround times by the different components – weekday specimens**


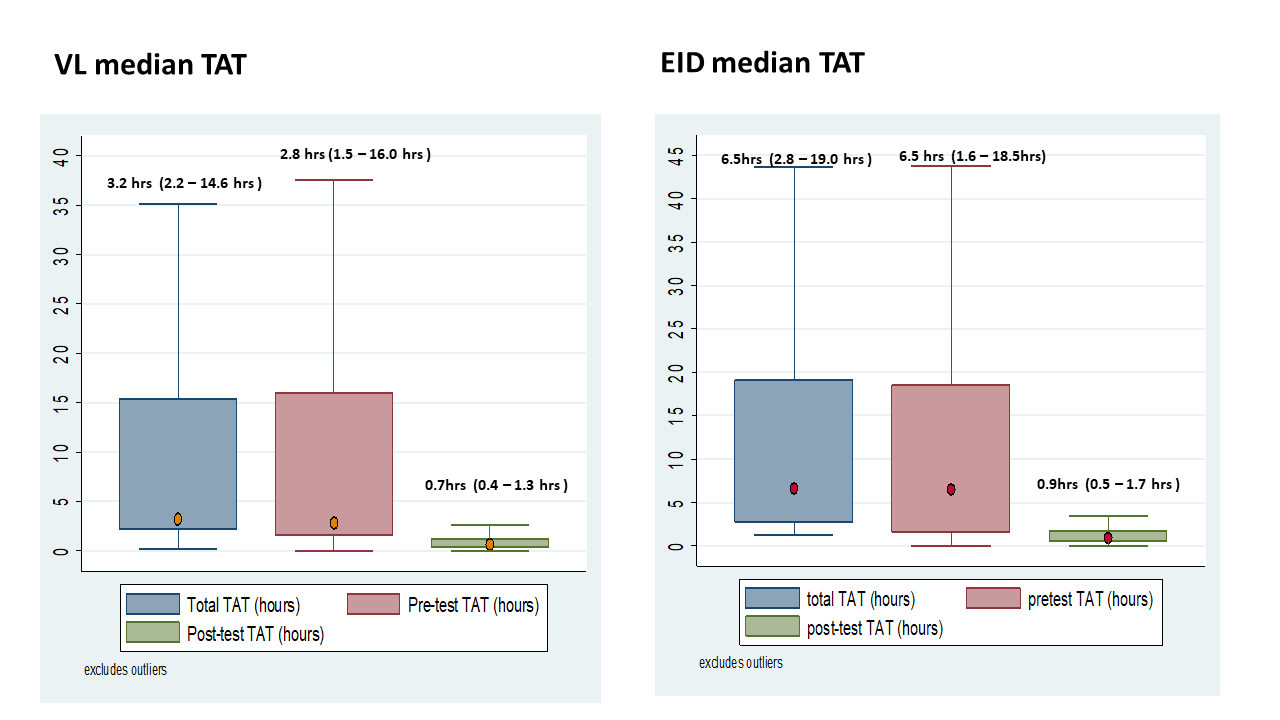


**Figure S3: Comparison of pre-test POC and CLT pre-test periods – weekday specimens**


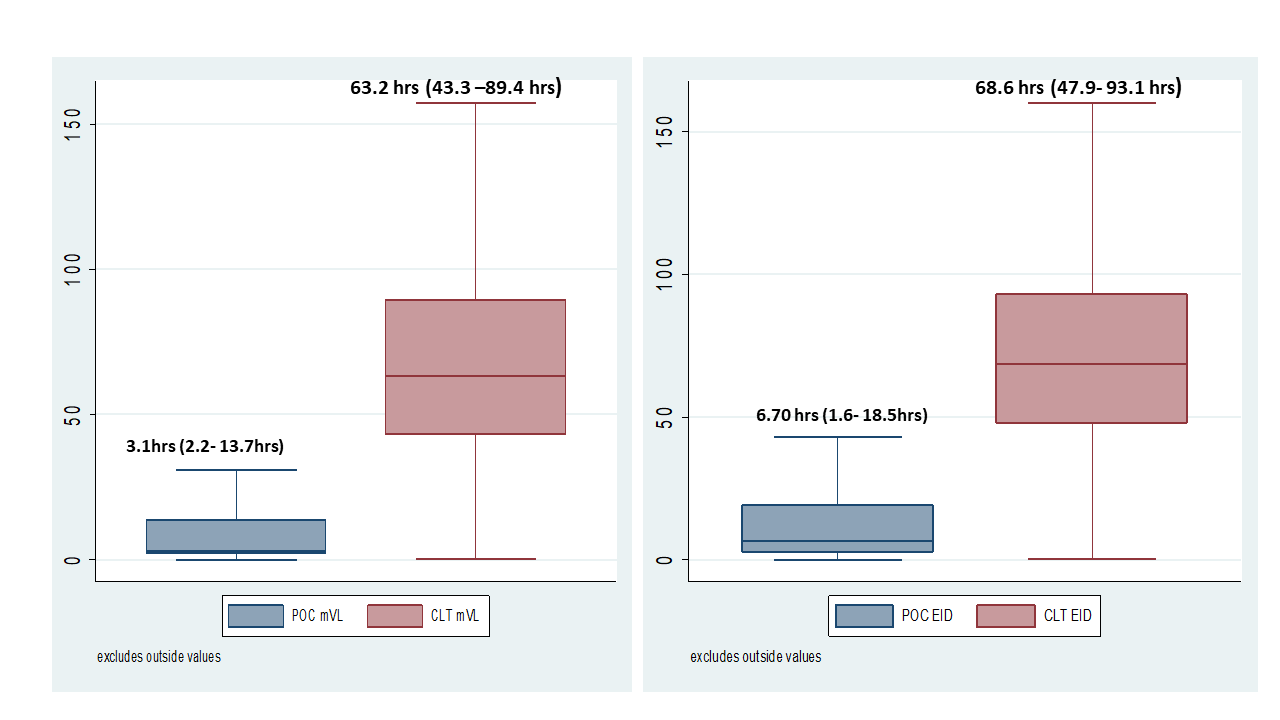

Supplement: Supplementary file 1 — Supplementary Document SD1. Supplementary methods Figure S1. Turn‐around times for weekday and weekend specimens. Figure S2. Turnaround times by the different components – weekday specimens. Figure S3. Comparison of pre‐test POC and CLT pre‐test periods – weekday specimens. [file JIA2-23-e25487-s001.docx]
