# Supplementary material for: Point‐of‐care HIV maternal viral load and early infant diagnosis testing around time of delivery at tertiary obstetric units in South Africa: a prospective study of coverage, results return and turn‐around times
Source: J Int AIDS Soc. 2020 Apr 23;23(4):e25487. doi: 10.1002/jia2.25487 (PMC7180267; doi:10.1002/jia2.25487)
Supplement: Supplementary file 2 — Table S1. Differences in POC instruments and staff per site [file JIA2-23-e25487-s002.docx]

**Supplementary information: Detailed data collection methods**

*Specimen collection*

Following verbal consent and pretest counselling, two samples were collected from each pregnant WLHIV and HIV-exposed infant. For pregnant WLHIV, two 4ml EDTA tubes were requested – one for sending to the routine centralised laboratory and the other for POC testing. For infants, two microtainer EDTA tubes (each with 250µl blood) for parallel POC testing and CLT were requested. Alternatively, one 250μl EDTA specimen for POC testing and one dried blood spot card, with at least three 70μl spots, for CLT were requested. Specimens were collected by doctors and nurses as part of their routine duties. Non-study patients could access HIV EID and VL POC testing where clinically indicated and these were labelled ‘miscellaneous’.

*POC testing*

POC testing was conducted by a POC operator working in a designated POC testing room. The location of the POC testing room varied at each site and was dependent on available space closest to the labour ward. At two of the TOU (Johannesburg region F and Tshwane) side-rooms located within the labour ward complex were utilized. At Johannesburg region D a side-room opposite one of the post-natal wards was used and at Johannesburg region B an established satellite laboratory located in the same building as the postnatal wards was used. All POC testing room were within a 100m walking distance of both labour and post-natal wards.

POC operators were dedicated staff, hired and trained as part of the implementation study. At the Johannesburg region D and F TOUs, POC operators were professional nurses while at the remaining two sites, they were an enrolled nurse and a laboratory technician respectively. Operators were supported and supervised by a study coordinator and a management team. If operators were absent, the study coordinator and data coordinator, a registered nurse and a laboratory scientist respectively, stood in for them. While specimen collection took place throughout the week including weekends and after-hours at all but one TOU (Johannesburg Region B), testing took place during working hours - 08h00 to 16h00 - on weekdays only. During the first three months of implementation, all specimens collected were processed and tested while afterwards only weekday specimens and those weekend specimens which had a reasonable probability of results being returned were tested. At two of the busier TOUs (Johannesburg region B and D), two dedicated counsellors hired by the study assisted with return of results and post-test counselling before discharge.

*POC testing procedures*

For POC VL testing, Xpert™ HIV-1 VL was used while POC EID testing was conducted using either the Xpert™ HIV-1 Qual or the m-PIMA HIV-1/2 Detect assays. Upon receiving appropriate samples, POC operators entered specimen details into the instruments’ information management system and tested the specimens according to manufacturers’ specifications and sample volumes. For plasma VL this meant centrifuging specimens at 3.3 rounds per minute (rpm) for 15 minutes before testing could be conducted. Run times of both the Xpert™ HIV-1 VL and Qual assays was 90 minutes whereas on the m-PIMA HIV-1/2 Detect assay was 52 minutes. All Xpert™ instruments used were four-modular, permitting simultaneous VL and EID testing, whereas the m-PIMA instruments allowed only one sample to be tested at a time. The number of instruments per site was determined by projected patient volume to ensure sufficient testing capacity per site to prevent unnecessary testing delays and are presented in Supplementary Table S1 below.

Infant specimens were tested on either the Xpert™ or m-PIMA depending on availability of a free module. However, as m-PIMA testing was only introduced in October 2018 less samples were tested using this assay. Mothers and infants whose results were reported as error, invalid or no result had the test repeated on the same sample and if there was still no conclusive result, a second sample was collected as soon as possible after the result. Infants with a positive POC EID result were repeat tested on the same assay using the same sample to ensure reproducibility, and a second sample was requested for confirmatory testing on the alternate EID assay.

**Table S1: Differences in POC instruments and staff per site**

|  | **Jhb B** | **Jhb D** | **Jhb F** | **Tshwane** |
| --- | --- | --- | --- | --- |
| **POCT implemented (Established/New)** |  |  |  |  |
| HIV PCR for EID | Established | Established | New | New |
| HIV VL for mothers at delivery | New | New | New | New |
| **Instruments installed** |  |  |  |  |
| Xpert® | 3 | 2 | 1 | 1 |
| m-PIMA EID | 2 | 2 | 1 | 0 |
| m-PIMA VL | 3 | 2 | 0 | 0 |
| **Site-based study personnel** |  |  |  |  |
| POCT operator | 1 (laboratory technician) | 1 (nurse) | 1 (nurse) | 1 (nurse) |
| Counsellor | 5^*^ | 1 | 0 | 0 |
| Co-investigator | 1 | 0 | 0 | 0 |
| **Weekend samples** | Never collected | Testing of weekend samples stopped on 27 August 2018 | Testing of weekend samples stopped on 27 August 2018 | Testing of weekend samples stopped on 27 August 2018 |

**^*^**One was appointed for the study. Others were routine staff in place at the start of the study

*Quality assurance around POC testing*

POC operators were trained to operate instruments according to good laboratory practice by the instrument manufacturers. Operators were provided with standard operating procedures including visual aids for reference. During implementation, site visits from the manufactures were arranged as needed. The project co-ordinator visited each site weekly and was available for telephonic support. Validation of every instrument was performed prior to implementation. This involved the reference laboratory sending blinded samples, for testing by the POC operators on both EID and VL instruments, and evaluating the results obtained. At three different time points during implementation, the reference laboratory provided blinded samples for POC operators to test and return results as part of external quality assurance (EQA) activities.

*Additional data analysis methods*

Sensitivity analyses for coverage of testing - using a denominator that excluded births to HIV-positive women which took place during the weekends and public holidays - was done to determine the estimated minimum as well as the maximum coverage that could have been attained. Minimum coverage was determined as the number of WLHIV tested/ the number of HIV-exposed infants tested divided by the total number of infants delivered to WLHIV during the study period. The maximum coverage was determined as the number of WLHIV tested/ number of HIV-exposed infants tested divided by the total number of infants delivered to WLHIV during the study period but excluding public holidays and weekends when specimens collected would not have been tested. For the Poisson regression models used to determine factors independently associated with results not being returned, all available variables were included in the multivariable models *a priori*. Because timing of specimen collection varied across TOUs, we allowed for interaction between timing of specimen collection – weekend, weekday after-hours and weekday workhours- with TOU in both infant and maternal results return models. The sign-test was used to test the hypothesis that the median turn-around time of maternal VL load results was equal to the median turn-around time of EID tests
