# Supplementary material for: Point‐of‐care HIV maternal viral load and early infant diagnosis testing around time of delivery at tertiary obstetric units in South Africa: a prospective study of coverage, results return and turn‐around times
Source: J Int AIDS Soc. 2020 Apr 23;23(4):e25487. doi: 10.1002/jia2.25487 (PMC7180267; doi:10.1002/jia2.25487)
Supplement: Supplementary file 3 — Table S2. Data sources and management for point‐of‐care implementation outcomes evaluation Table S3. (A) Performance of POC mVL testing compared to CTL. (B) Overall Performance of POC EID testing compared to CTL. (C) Performance of POC EID testing (Xpert HIV‐1/2 Qualitative) compared to CTL (D) Performance of POC EID testing (q‐HIV1/2 Detect) compared to CTL [file JIA2-23-e25487-s003.docx]

**Table S2: Data sources and management for point of care implementation outcomes evaluation**

| **Data source** | **Description** |
| --- | --- |
| Specimen request form and study specific REDCap® database | - Used by POC operators to capture data from the specimen request form - Each entry was identified with a number assigned at specimen receipt - Data elements on the specimen request form were first name, surname, hospital number, a unique barcode, ward, date and time of specimen collection and test being requested - No other data elements such as maternal ART status, duration on ART, |
| POC instrument information system | - System recorded date and time of test, cartridge batch and lot numbers and test results. - Data from system were exported in real-time by an application programming interface into the REDCap® database - Test result was linked to patient details using the unique REDCap® ID. |
| Result print-outs | - These were printed out by POC operators - Printed out as two hard copies of results for return to patient. - First copy was filed in the patient’s folder at bedside - Second copy used to capture date and time return of results took place - Date and time details on second were returned to POC operator for capturing into the REDCap® database. |
| Centralised laboratory information system | - Details on parallel VL and EID specimens sent to the centralised laboratory were obtained from centralised laboratory information system through weekly downloads - These details were also captured entered into REDCap® database using the name, surname, hospital number and/or laboratory request form barcode to link to POC results. |
| District Health Information System (DHIS) monthly reports | - Monthly reports of number of live-births to WLHIV were obtained from DHIS by POC operators - These monthly statistics were send to the study coordinator for collation and use by team |

**Table S3A: Performance of POC mVL testing compared to CTL**

|  | **Undetectable/ Detectable** | **VL>50 copies/ml** | **VL 50- 1000 copies** | **VL>1000** |
| --- | --- | --- | --- | --- |
| % Agreement  (95% CI) | 78.86% (77.17- 80.47%) | 90.60% (89.34- 1.72%) | 87.97 %( 86.59- 89.22%) | 98.17% (97.54- 98.64%) |
| Se  (95% CI) | 81.80% (80.24- 83.36%) | 84.39% (82.93- 85.86%) | 53.93% (51.92- 55.94%) | 98.78% (98.34- 99.22%) |
| Sp  (95% CI) | 75.31% (73.56 -77.05%) | 94.43% (93.51- 95.36%) | 96.20% (95.43- 96.97%) | 98.01 % (97.45- 98.58%) |
| PPV  (95% CI) | 80.12% (78.51- 81.73%) | 90.33% (89.14- 91.53%) | 77.43% (75.74- 79.12%) | 92.93% (91.89- 93.96%) |
| NPV  (95% CI) | 77.27% (75.58- 78.97%) | 90.75% (89.58- 91.92%) | 89.62% (88.39- 90.85%) | 99.67% (99.44- 99.90%) |

Limits of agreement (log_10_ VL) = -0.549 - 0.798; Mean differences (logVL) for detectable virus = 0.124 (95% CI 0.104 - 0.145)

**Table S3B: Overall Performance of POC EID testing compared to CTL**

| **POC/ CLT** | **Positive CLT** | **Negative CLT** | **Indeterminate on CLT** | **Error on CLT** | **Invalid on CLT** | **No result on CLT** | **Rejected on CLT** |
| --- | --- | --- | --- | --- | --- | --- | --- |
| **Positive POC EID** | 66 | 5 | 3 | 0 | 0 | 9 | 1 |
| **Negative POC EID** | 5 | 4005 | 5 | 2 | 4 | 210 | 44 |
| **Error on POC** | 1 | 126 | 0 | 0 | 0 | 3 | 1 |
| **Invalid on POC** | 1 | 526 | 0 | 0 | 1 | 47 | 6 |
| **No result on POC** | 0 | 2 | 0 | 0 | 0 | 0 | 1 |

Agreement/ accuracy = 99.51% (95% CI 99.27- 99.67); Sensitivity 90.41% (95% CI 89.60- 91.22%); Specificity 99.64% (95% CI 99.48- 99.80%); Positive predictive value 78.57% (95% CI 77.44- 79.70%); Negative predictive value 99.86% (95% CI 99.76- 99.96%)

**Table S3C: Performance of POC EID testing (Xpert HIV-1/2 Qualitative) compared to CTL**

| CLT EIDpos

POC EIDpos| 0 1 | Total

-----------+----------------------+----------

0 | 4,163 7 | 4,170

1 | 18 60 | 78

-----------+----------------------+----------

Total | 4,181 67 | 4,248

True D defined as CLT EID pos = 1 [95% Conf. Inter.]

-------------------------------------------------------------------------

Sensitivity Pr( +| D) 89.55% 88.63% 90.47%

Specificity Pr( -|~D) 99.57% 99.37% 99.77%

Positive predictive value Pr( D| +) 76.92% 75.66% 78.19%

Negative predictive value Pr(~D| -) 99.83% 99.71% 99.96%

-------------------------------------------------------------------------

Prevalence Pr(D) 1.58% 1.20% 1.95%

-------------------------------------------------------------------------

Agreement/ accuracy 99.41% (95% CI 99.13-99.60)

**Table S3D: Performance of POC EID testing (q-HIV1/2 Detect ) compared to CTL**

| CLT EIDpos

POC EIDpos| 0 1 | Total

-----------+----------------------+----------

0 | 820 0 | 820

1 | 0 6 | 6

-----------+----------------------+----------

Total | 820 6 | 826

True D defined as CLT EID positive ~= 1 [95% Conf. Inter.]

-------------------------------------------------------------------------

Sensitivity Pr( +| D) 100.00% 100.00% 100.00%

Specificity Pr( -|~D) 100.00% 100.00% 100.00%

Positive predictive value Pr( D| +) 100.00% 100.00% 100.00%

Negative predictive value Pr(~D| -) 100.00% 100.00% 100.00%

-------------------------------------------------------------------------

Prevalence Pr(D) 0.73% 0.15% 1.31%

-------------------------------------------------------------------------

Agreement/ accuracy 100%
